# Supplementary material for: Closing yield gaps for rice self-sufficiency in China
Source: Nat Commun. 2019 Apr 12;10:1725. doi: 10.1038/s41467-019-09447-9 (PMC6461608; doi:10.1038/s41467-019-09447-9)
Supplement: Supplementary file 1 — Supplementary Information [file 41467_2019_9447_MOESM1_ESM.pdf]

Supplementary Information

Closing yield gaps for rice self-sufficiency in China

Deng *et al.*

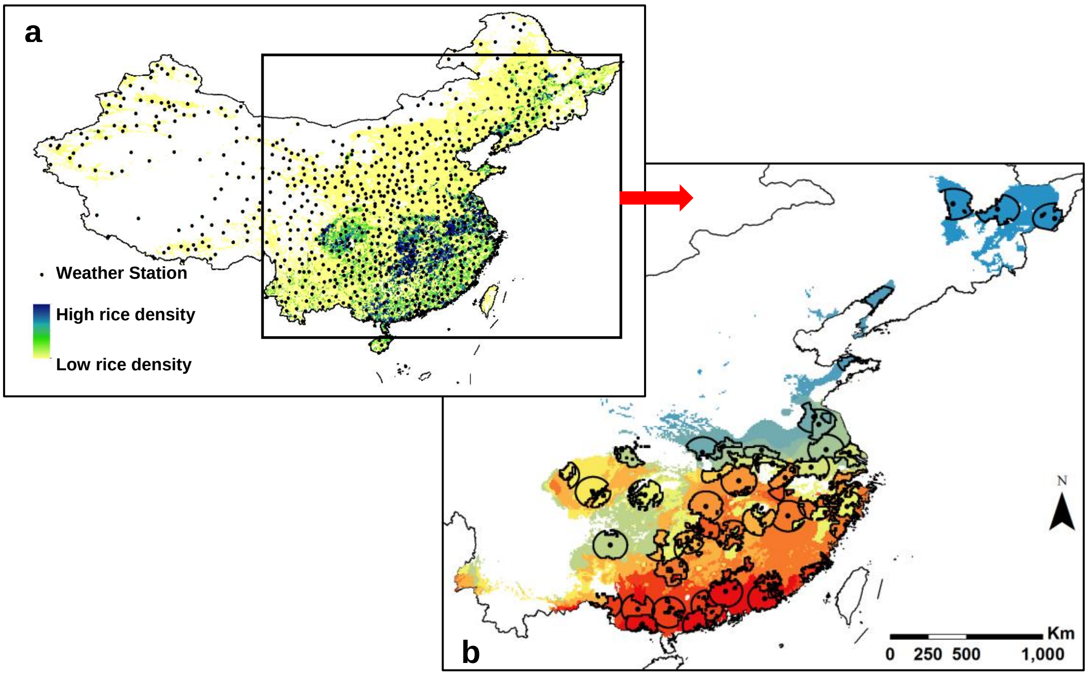

Supplementary Figure 1. Selected reference weather stations (RWS) and climate zones (CZ). a, Locations of 836 weather stations (dots) from the China Meteorological Administration weather database (National Meteorological Information Center, http://data.cma.cn), and rice harvested area density (SPAM map)[1]. **b**, Selected RWS (black dots), borders of RWS buffers (black lines), and CZ (different colors) in China. In total, 50 RWS were selected in northeast, north, central, and south China, accounting for 48% of national rice harvested area within RWS buffer zones. The 50 selected RWS are located in 16 CZ, which, in turn, account for 85% of national rice harvested area. Source data are provided as a Source Data file.

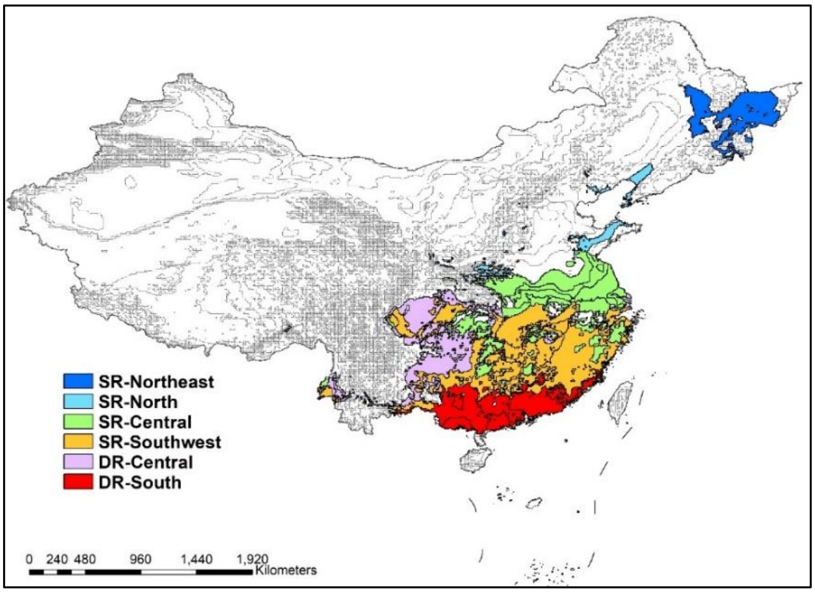

Supplementary Figure 2. Six rice production regions and dominant rice cropping system in each region. Black lines in the background outline the 139 climate zones in China; of these, 16 climate zones covering 85% of rice growing areas are colored, with each color corresponding to one of the six production regions delineated for selection of dominant management practices and cultivars. For the purpose of simulating potential yield, crop management practices, including rice cropping system, cultivar and sowing date, are considered to be similar within each region, which may contain from 1 to 5 climate zones. Source data are provided as a Source Data file.

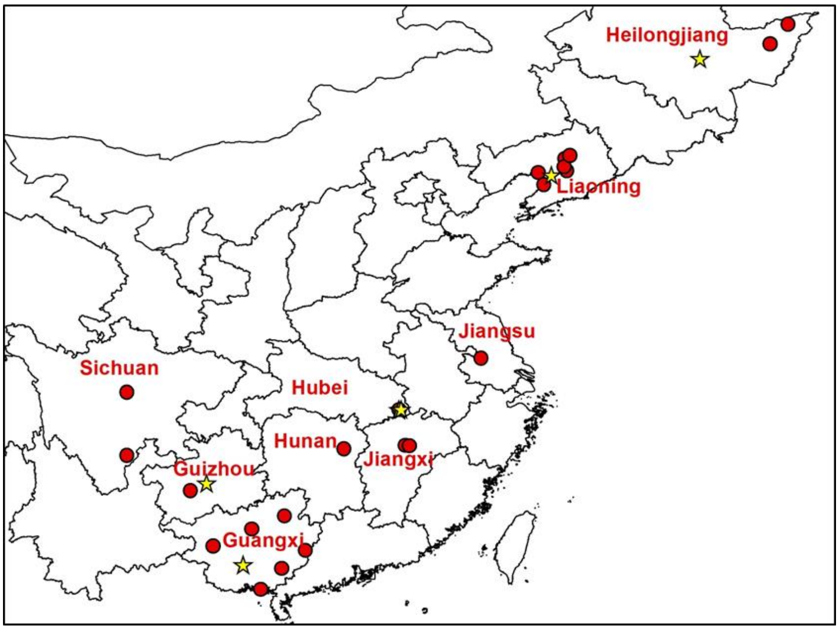

Supplementary Figure 3. Experimental sites for ORYZA model calibration and validation. Yellow stars are the calibration and validation 1 experimental sites (n=6), and red points are the validation 2 experimental sites (n=23). The related names of provinces are shown in red. Source data are provided as a Source Data file.

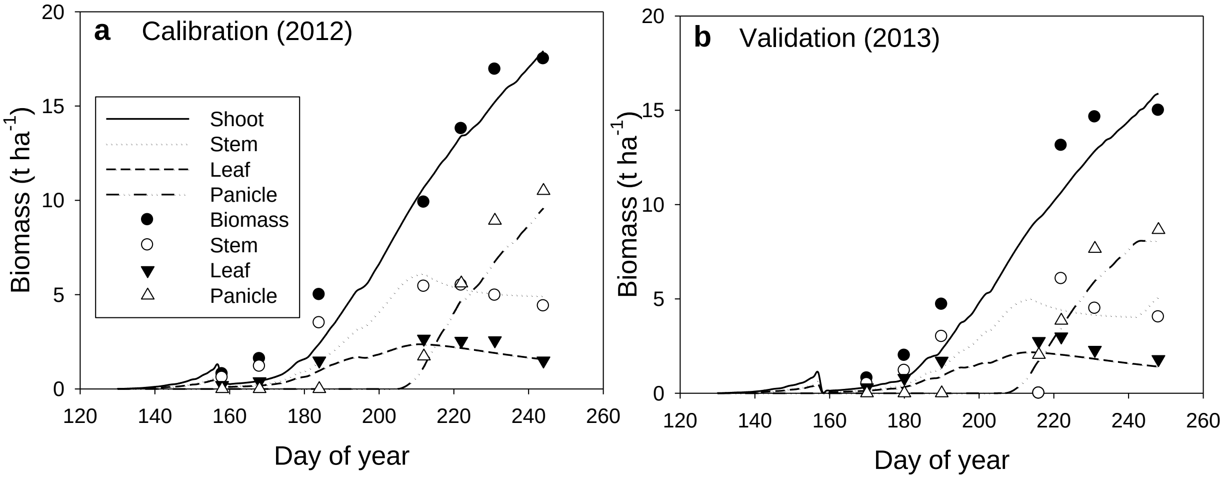

Supplementary Figure 4. ORYZA rice model calibration results for rice cultivar Huanghuazhan. Comparison of (**a**) calibrated (2012) and (**b**) validated (2013) results of rice cultivar Huanghuazhan by using ORYZA rice model. Simulated (lines) and observed values (symbols) are shown for shoot (i.e., aboveground biomass), green leaf, stem, and panicle biomass. Source data are provided as a Source Data file.

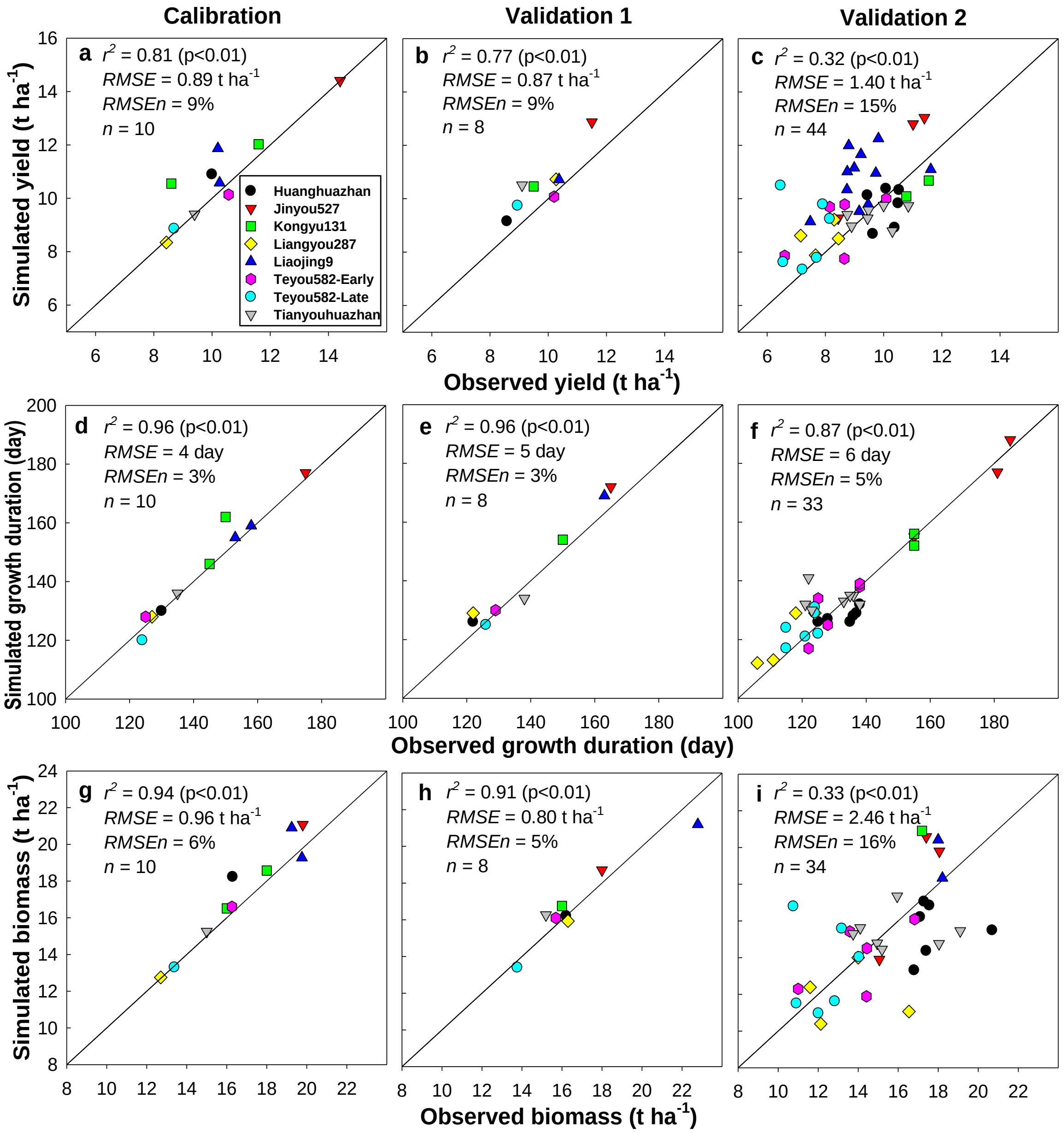

Supplementary Figure 5. ORYZA rice model calibration and validation. Comparison of calibrated versus observed grain yield (**a**), aboveground biomass (**d**), and growth duration (**g**), validated dataset 1 (data were from same experimental sites or same paper as calibration dataset) versus observed grain yield (**b**), shoot biomass (**e**) and growth duration (**h**) and validated dataset 2 (data were from different experimental sites or different paper as calibration dataset) versus observed grain yield (**c**), shoot biomass (**f**) and growth duration (**i**) by using calibrated ORYZA rice model. Symbols with same colors represent the same cultivar grown in different years or sites. Line in each figure is the 1 to 1 line. Data were collected from experimental site (Huanghuazhan in central China) and from high-yield experiments in published papers. Source data are provided as a Source Data file.

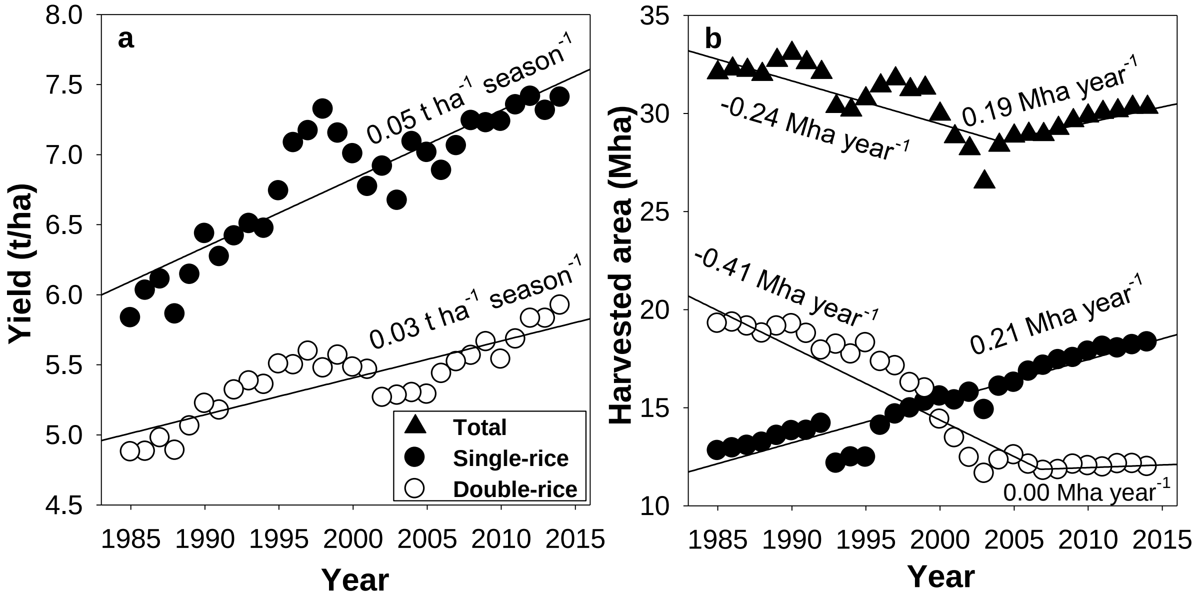

Supplementary Figure 6. Trends in national average rice yield (a) and harvested area (b) in China. The data were disaggregated by total- (single and double), single- and double-rice crop production systems. Note that yields for double-rice are the average for the two rice crops grown each year in the same field so that total rice output per hectare is twice the values shown. Data were obtained from NBSC[2]. Mha: million hectares. Source data are provided as a Source Data file.

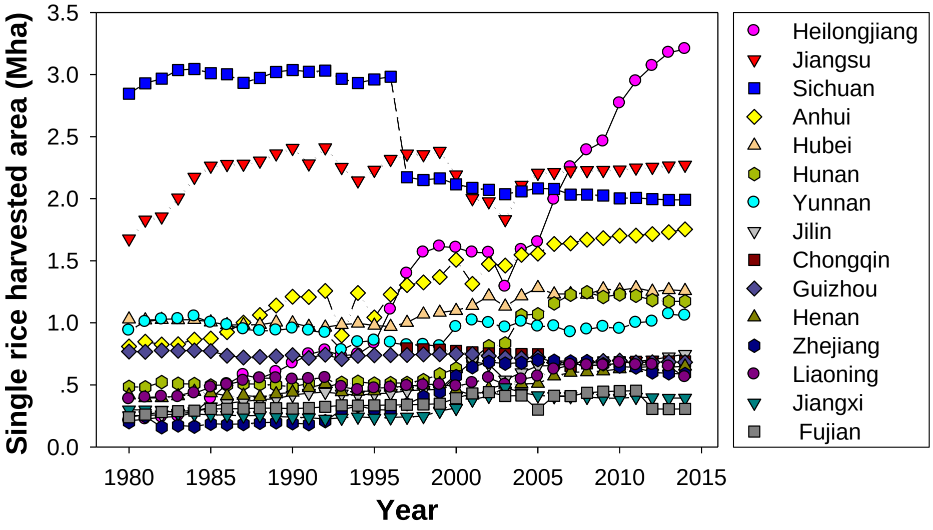

Supplementary Figure 7. Trends in rice harvested area of single-rice by province since 1980. Data were obtained from NBSC[2]. Mha: million hectares. Source data are provided as a Source Data file.

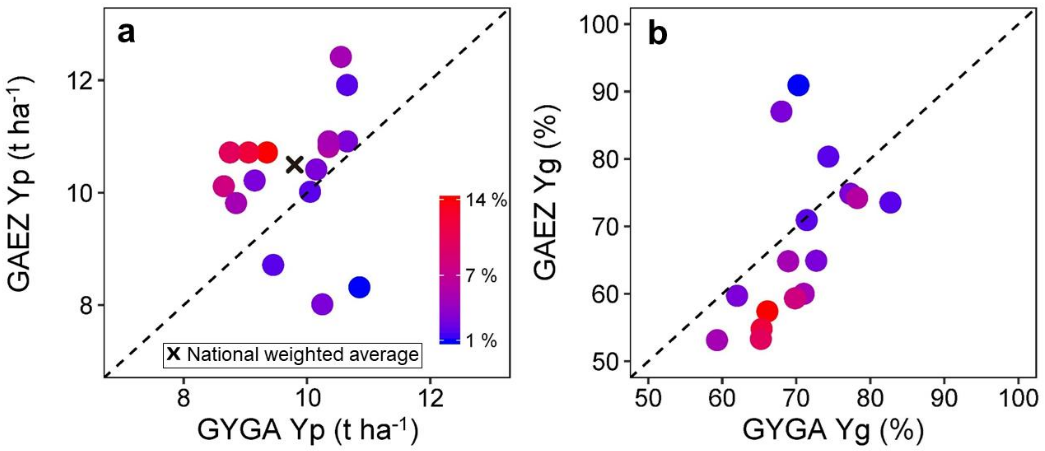

Supplementary Figure 8. Comparison of Yp and Yg between GYGA and GAEZ. Comparison of (**a**) Yp and (**b**) Yg (Ya/Yp %) estimated by GYGA (Global Yield Gap Atlas) and GAEZ (Global Agro-ecological Zones)[3] at CZ level by weighting irrigated rice harvested area based on the SPAM map. Each observation represents one of the 16 major rice-growing CZs, and the different symbol colors represent the percentage of national rice harvested area contained within each CZ. Yp: potential yield; Ya: farm yield; Yg: yield gap. Source data are provided as a Source Data file.

Supplementary Table 1. Location for the reference weather stations used in this study

RWS ID	County, Province	Longitude (º)	Latitude (º)	Elevation (m)	Cropping system*
1	Xiangfan, Hubei	112.2	32.0	70	Single-rice
2	Muyang, Jiangsu	118.8	34.1	9	Single-rice
3	Tongbai, Henan	113.4	32.4	149	Single-rice
4	Huaiyin, Jiangsu	119.0	33.7	15	Single-rice
5	Gushi, Henan	115.7	32.2	58	Single-rice
6	Dantu, Jiangsu	119.5	32.2	29	Single-rice
7	Dawu, Hubei	114.1	31.6	72	Single-rice
8	Nantong, Jiangsu	120.9	32.0	6	Single-rice
9	Huoshan, Anhui	116.3	31.4	73	Single-rice
10	Wuhu, Anhui	118.6	31.2	20	Single-rice
11	Qianjiang, Chongqing	108.8	29.5	609	Single-rice
12	Chengbu, Hunan	111.5	26.4	476	Single-rice
13	Xianju, Zhejiang	120.7	28.9	52	Single-rice
14	Wanyuan, Sichuan	108.0	32.1	674	Single-rice
15	Guiyang, Guizhou	106.7	26.6	1074	Single-rice
16	Wenjiang, Sichuan	103.8	30.7	541	Single-rice
17	Dazu, Chongqing	105.7	29.7	394	Single-rice
18	Jingan, Jiangxi	115.4	28.9	80	Single-rice
19	Haicheng, Liaoning	122.7	40.9	27	Single-rice
20	Fushan, Shandong	121.3	37.5	34	Single-rice
21	Suihua, Heilongjiang	127.0	46.6	180	Single-rice
22	Yilan, Heilongjiang	129.6	46.3	101	Single-rice
23	Hulin, Heilongjiang	133.0	45.8	104	Single-rice
24	Tianmen, Hubei	113.2	30.7	35	Double-rice
25	Jingzhou, Hunan	109.7	26.6	321	Double-rice
26	Shaoyang, Hunan	111.3	27.0	278	Double-rice
27	Cixi, Zhejiang	121.3	30.2	8	Double-rice
28	Jinhua, Zhejiang	119.7	29.1	65	Double-rice
29	Zhangshu, Jiangxi	115.6	28.1	30	Double-rice
30	Jiangxia, Hubei	114.3	30.4	38	Double-rice
31	Yuanjiang, Hunan	112.4	28.9	36	Double-rice
32	Anqing, Anhui	117.1	30.5	20	Double-rice
33	Hangzhou, Zhejiang	120.2	30.2	43	Double-rice
34	Lianhua, Jiangxi	114.0	27.1	181	Double-rice
35	Guilin, Guangxi	110.3	25.1	172	Double-rice
36	Guixi, Jiangxi	117.2	28.3	52	Double-rice
37	Quzhou, Zhejiang	118.9	29.0	67	Double-rice
38	Ruian, Zhejiang	120.6	27.8	10	Double-rice
39	Shuangfeng, Hunan	112.2	27.5	98	Double-rice
40	Dongzhi, Anhui	117.0	30.1	23	Double-rice
41	Nanxiong, Guangdong	114.3	25.1	135	Double-rice
42	Xianyou, Fujian	118.7	25.4	77	Double-rice
43	Meixian, Guangdong	116.1	24.3	89	Double-rice
44	Gaoyao, Guangdong	112.5	23.1	12	Double-rice
45	Nanning, Guangxi	108.4	22.8	74	Double-rice
46	Yulin, Guangxi	110.2	22.7	85	Double-rice
47	Fogang, Guangdong	113.5	23.9	68	Double-rice
48	Jiexi, Guangdong	115.8	23.4	42	Double-rice
49	Qinzhou, Guangxi	108.6	22.0	6	Double-rice
50	Yangjiang, Guangdong	112.0	21.9	22	Double-rice

*Dominant rice cropping system, with either one (single-) or two (double-) rice crops per year. Dominant cropping system (single- or double-rice) identified for each RWS buffer was used as the basis for simulation of potential yield and estimation of yield gaps.

Supplementary Table 2. Crop management information for each of the six rice-production regions and data source for calibration and validation of the ORYZA rice model

Calibration infomation	Kongyu 131	Liaojing9	Huang huazhan	Jinyou 527	Liang you287	Tianyou huazhan	Teyou 582	Teyou 582
Region	Northeast	North	Central	South-west	Central	Central	Southern	Southern
Cropping system	Single	Single	Single	Single	Double-early	Double-late	Double-early	Double-late
Growth duration (d)	130-145	150-160	115-125	155-170	115-130	115-135	115-135	105-120
Calibration and Validation 1								
Site:Province (county)	Heilong jiang (Yilan)	Liaoning (Haicheng)	Hubei (Wuxue)	Guizhou (Guiyang)	Hubei (Wuxue)	Hubei (Wuxue)	Guangxi (Nanning)	Guangxi (Nanning)
Location (Long., lat. Elevation)	129.6, 46.3, 101m	122.7, 40.9, 27m	115.03, 30.3, 198m	106.7, 26.6, 1074m	114.3, 30.4, 41m	114.3, 30.4, 41m	108.4, 22.8, 74m	114.3, 30.4, 41m
Calibration year	2010, 2012	2006-2007, 2009	2012	2008	2014	2013	2010	2010
Validation 1 year	2011	2006-2007	2013	2009	2013	2012	2011	2011
Reference	(4-5)	(8-9)	Experiment	(18)	(17, 20)	(17, 22)	(26)	(26)
Validation 2								
Station site: province (country)	Heilong jiang (Fujing)	Liaoning (Dawu, Sunjiatun, Liaoyang, Doingling, Gaixian, Shengyang)	Hunan (Changsha), Hubei (Wuxue)	Guizhou (Anshun), Sichuan (Xichang, Ya'an)	Hubei (Wuxue)	Jiangxi (Jinxian, Nanchang), Jiangsu (Yangzhou), Hubei (Wuxue)	Guangxi (Guilin, Hechi, Wuzhou, Baise, Yulin)	Guangxi (Wuzhou, Hepu, Baise, Nanning)
Year	2007, 2010	2005-2007	2012-2015	2008-2009	2013-2015	2008, 2010, 2013-2015	2010-2011	2010-2012
Datasets reference	(6-7)	(9-15)	(16-17)	(18-19)	(17, 21-22)	(17, 21, 23-25)	(26)	(26-27)

Supplementary Table 3. Modifications and calibrations to key parameters in ORYZA rice model

Calibration information	Kongyu 131	Liaojing9	Huang huazhan	Jinyou 527	Liangyou 287	Tianyou Huazhan	Teyou 582	Teyou 582
TMD	42.0	42.0	42.6	42.0	42.6	42.6	42.6	42.6
TOD	30.00	30.00	31.17	30.00	31.17	31.17	31.17	31.17
COLDEAD (d)	20	10	5	10	10	5	10	5
DVRJ	0.0011770	0.0009228	0.0007659	0.0007600	0.0015291	0.0007553	0.0010964	0.0011770
DVRI	0.0007576	0.0007576	0.0008076	0.0007580	0.0007576	0.0007576	0.0007576	0.0007576
DVRP	0.0005697	0.0007007	0.0007698	0.0006640	0.0008615	0.0007692	0.0006465	0.0005697
DVRR	0.0016832	0.0015550	0.0016842	0.0014910	0.0020298	0.0015905	0.0019714	0.0016832
FSTR	0.35	0.10	0.20	0.33	0.36	0.15	0.38	0.20
FSH0.00	0.50	0.50	0.60	0.50	0.70	0.50	0.70	0.50
FSH0.43	0.65	0.65	0.72	0.50	0.75	0.65	0.75	0.50
FSH1.00	1.00	1.00	1.00	1.00	1.00	1.00	1.00	1.00
FLV0.00	0.60	0.30	0.50	0.50	0.50	0.50	0.50	0.50
FLV0.44	0.50	0.30	0.40	0.32	0.40	0.40	0.50	0.40
FLV0.64	0.40	0.40	0.40	0.37	0.40	0.30	0.40	0.30
FLV0.90	0.30	0.40	0.30	0.32	0.30	0.30	0.30	0.30
FLV1.00	0.30	0.10	0.00	0.00	0.00	0.20	0.00	0.20
FLV1.16	0.00	0.20	0.00	0.00	0.00	0.00	0.00	0.00
FLV1.49	0.00	0.00	0.00	0.00	0.00	0.00	0.00	0.00
FST0.00	0.40	0.70	0.50	0.50	0.50	0.50	0.50	0.50
FST0.44	0.50	0.70	0.60	0.68	0.60	0.60	0.50	0.60
FST0.64	0.60	0.60	0.60	0.63	0.60	0.70	0.60	0.70
FST0.90	0.70	0.60	0.70	0.68	0.70	0.70	0.70	0.70
FST1.00	0.00	0.30	0.00	0.00	0.00	0.30	0.00	0.30
FST1.16	0.00	0.20	0.00	0.00	0.00	0.00	0.00	0.00
FST1.49	0.00	0.00	0.00	0.00	0.00	0.00	0.00	0.00
FSO0.00	0.00	0.00	0.00	0.00	0.00	0.00	0.00	0.00
FSO0.44	0.00	0.00	0.00	0.00	0.00	0.00	0.00	0.00
FSO0.64	0.00	0.00	0.00	0.00	0.00	0.00	0.00	0.00
FSO0.90	0.00	0.00	0.00	0.00	0.00	0.00	0.00	0.00
FSO1.00	0.70	0.60	1.00	1.00	1.00	0.50	1.00	0.50
FSO1.16	1.00	0.60	1.00	1.00	1.00	1.00	1.00	1.00
FSO1.49	1.00	1.00	1.00	1.00	1.00	1.00	1.00	1.00

Supplementary Table 4. Description of the calibrated model parameters in Supplementary Table 3

Parameter	Description	Parameter	Description
TMD	Maximum temperature for development (^{o}C)	FLV1.16	Fraction shoot dry matter partitioned to leaves at DVS = 1.16
TOD	Optimum temperature for development (^{o}C)	FLV1.49	Fraction shoot dry matter partitioned to leaves at DVS = 1.49
COLDEAD	Consecutive number of days below COLDMIN that crop dies	FSTR	Fraction of carbohydrates allocated to stems that is stored as reserves
DVRJ	Development rate in juvenile phase ($^{o}Cd^{-1}$)	FST0.00	Fraction shoot dry matter partitioned to stems at DVS = 0
DVRI	Development rate in photoperiod-sensitive phase ($^{o}Cd^{-1}$)	FST0.44	Fraction shoot dry matter partitioned to stems at DVS = 0.44
DVRP	Development rate in panicle development ($^{o}Cd^{-1}$)	FST0.64	Fraction shoot dry matter partitioned to stems at DVS = 0.64
DVRR	Development rate in reproductive phase ($^{o}Cd^{-1}$)	FST0.90	Fraction shoot dry matter partitioned to stems at DVS = 0.9
DVS	Development stage (DVS = 0: sowing; 0.4: photoperiod-sensitive phase; 0.65: panicle initiation; 1: flowering; 2: physiological maturity)	FST1.00	Fraction shoot dry matter partitioned to stems at DVS = 1
FSTR	Fraction of carbohydrates allocated to stems that is stored as reserves (-)	FST1.16	Fraction shoot dry matter partitioned to stems at DVS = 1.16
FSH0.00	Fraction total dry matter partitioned to shoot at DVS = 0	FST1.49	Fraction shoot dry matter partitioned to stems at DVS = 1.49
FSH0.43	Fraction total dry matter partitioned to shoot at DVS = 0.43	FSO0.00	Fraction shoot dry matter partitioned to panicles at DVS = 0
FSH1.00	Fraction total dry matter partitioned to shoot at DVS = 1	FSO0.44	Fraction shoot dry matter partitioned to panicles at DVS = 0.44
FLV0.00	Fraction shoot dry matter partitioned to leaves at DVS = 0	FSO0.64	Fraction shoot dry matter partitioned to panicles at DVS = 0.64
FLV0.44	Fraction shoot dry matter partitioned to leaves at DVS = 0.44	FSO0.90	Fraction shoot dry matter partitioned to panicles at DVS = 0.9
FLV0.64	Fraction shoot dry matter partitioned to leaves at DVS = 0.64	FSO1.00	Fraction shoot dry matter partitioned to panicles at DVS = 1
FLV0.90	Fraction shoot dry matter partitioned to leaves at DVS = 0.9	FSO1.16	Fraction shoot dry matter partitioned to panicles at DVS = 1.16
FLV1.00	Fraction shoot dry matter partitioned to leaves at DVS = 1	FSO1.49	Fraction shoot dry matter partitioned to panicles at DVS = 1.49

Supplementary Table 5. Summary of model calibration and validation efforts in previous studies simulating rice potential yield

Source	Study Case	Model	No. of sites for calibration	No. of cultivars for calibration	No. of validation data sets	Data source
Current study	China	ORYZA	6	7	52	Research papers and high-yield experiments (2005-2015)
(28)	China	ORYZA	NA[*]	Default[**]	NA	NA
(29)	China	ORYZA	19	25	69	CMA (1998-2009)[***]
(30)	China	MCWLA-Rice[****]	NA	NA	NA	Statistical yearbook
(31)	China	CERES-Rice	NA	NA	119	CMA (2003-2004)
(32)	Indonesia	ORYZA	3	1	21	High-yield experiments
(33)	Indonesia	ORYZA	1	Default	NA	NA
(34)	Indonesia	ORYZA	1	NA	NA	NA
(34)	Myanmar	ORYZA	1	NA	NA	NA
(33)	Philippines	ORYZA	1	Default	NA	NA
(33)	Thailand	ORYZA	1	Default	NA	NA
(34)	Thailand	ORYZA	1	NA	NA	NA
(35)	USA	ORYZA	10	2	NA	Experimental plots
(33)	Vietnam	ORYZA	1	Default	NA	NA
(34)	Vietnam	ORYZA	1	NA	NA	NA
(36)	African countries(8)	ORYZA	NA	NA	NA	NA

[*] NA: Not clearly mentioned in the paper.

[**] Default: default parameter value used in absence of calibration.

[***] In the MCWLA-Rice model, calibration is done by Bayesian probability inversion and the MCMC technique[37].

[****] CMA: China Meteorological Administration. Notice that, within the year range of the calibration database in the paper, 89% of the yields are before 2005.

Supplementary Table 6. Data source and uncertainties and quality control measures

Data	Data source	Uncertainties and/or limitations	Quality control measures
Weather data	CMA	Erroneous and missing data	Quality control following van Wart et al (2013)[28], Grassini et al. (2015)[38], and Global Yield Gap Atlas (http://www.yieldgap.org)
Average yield data, total production, and harvested area	NBSC	Possible yield bias at county level due to sample size	Adjusted to ensure consistency with provincial and national yield records
Experimental data for model calibration and validation	Original and published data	Not all experiments were not necessarily managed to reach potential yield or lack of detailed information for the experiments that reached potential yield	Selection of well management, high yield experiments for model calibration
Rice area distribution	SPAM	Crop area distribution around year 2005 may be outdated	Selection of high number of RWS across the entire rice producing region reduces the potential bias

Abbreviation: CMA: National Meteorological Information Center of the China Meteorological Administration; NBSC: National Bureau of Statistics of China.

Supplementary Table 7. Previous studies assessing rice production scenarios in China

Scenario	Projection Year	Self-sufficiency ratio	Reference
S1	2030	95%	Current study
S2	2030	100%	Current study
S3	2030	99%	Current study
S4	2030	100%	Current study
Ya increased by the yield trend from 1985 to 2014	2030	100%	(39)
Ya reaches to 80% Yield of ISSM*	2030	99%	(39)
Grain price fall + slightly import (2025)	2025	90%	(40)
A2**	2011-2040 (average)	100%	(41)
A2 + CO_2 fertilization	2011-2040 (average)	100%	(41)
B2	2011-2040 (average)	100%	(41)
B2 + CO_2 fertilization	2011-2040 (average)	100%	(41)
A2	2030	92%	(42)
A2 + tech	2030	95%	(42)
B2 + no tech	2030	100%	(42)
B2 + tech	2030	100%	(42)
Integration of biological mechanisms with economic mechanisms	2024	93%	(43)
-***	2027	97%	(44)
Projection of import and export in 2025	2025	100%	(45)

*ISSM is an integrated soil-crop system management including optimization of varieties, sowing dates, densities and advanced nutrient management.

** A2 and B2: IPCC SRES A2 and B2 scenarios in 2030/2050; “tech” means technology development.

*** No detailed information for the assessment scenario

Supplementary Table 8. Estimated potential total production if all rice farmers achieved yields that were 80% of potential exploitable production as estimated by protocols developed by GYGA or by GAEZ

Method	Exploitable production (MMT)	Increase over current production (206 MMT) (MMT)	Increase over production demand in 2030 (217 MMT) (MMT)
GYGA*	237	31	20
GAEZ	254	48	37

* GYGA: Global Yield Gap Atlas; GAEZ: Global Agro-Ecological Zones Model. Source data are provided as a Source Data file.

Supplementary References

1. You, L., U. Wood-Sichra, S. Fritz, Z. Guo, L. See, and J. Koo. 2014. Spatial Production Allocation Model (SPAM) 2005 v2.0. Available from http://mapspam.info
2. NBSC. *National Bureau of Statistics of China, China Statistical Yearbook.* (China Statistics Press, Beijing, 1980-2016).
3. IIASA/FAO. *Global Agro‐ecological Zones (GAEZ v3.0)*. (IIASA, Laxenburg, Austria and FAO, Rome, Italy, 2012).
4. Wang H., *Adaptive management of rice in cold region for high yield and high efficiency*, China Agricultural University, (2015).
5. Tian, Y. *et al.* Effects of water and nitrogen on growth,development and yield of rice in cold area of Northeast China. *Acta. Ecologica. Sin.* **34**, 6864-6871 (2014).
6. Dong, D., Huo, Z., Pan, X., Huang, Y. The effect of plant density on rice variety Kongyu131. *Agric. Tech.* **28**, 55-57 (2008).
7. Song, W. *et al.* Analysis on the characteristics of dry matter accumulation of rice with high yield in cold area. *Heilongjiang Agric. Sci.* **8**, 34-37 (2012).
8. Wang, Z. *Research on alternation rule about rice physiological and yield traits during cultivar replacement in Liaoning*, Chinese Academy of Agricultural Sciences, (2009).
9. Zhang, M., Na, Y. Regional Test of Rice New Variety of shenyang in 2009. *Horticult. Seed* **6**, 1-3 (2012).
10. Yu, H. *Studies on morphological and physiological characteristics of rice cultivars with high yield and good quality*, Shenyang Agricultural University, (2009).
11. Shao, G. *Studies on mechanism of good quality and high yield of liaojing rice varieties*, Shenyang Agricultural

University, (2007).

12. Shao, G. *et al.* Breeding and main yield characters of rice varieties of liaojing series. *J. Shenyang Agric. Univ.* **43**, 660-666 (2012).

13. Zhang, Y. *Study on grain yield and quality traits of rice in liaoning province*, Chinese Academy of Agricultural Sciences, (2010).

14. Zhang, X. Effects of high-yielding population in rice. *North. Rice* **41**, 7-11 (2011).

15. Wang, Z., Chen, Y., Wang, X., Zhang, C., Shang, W. Study on characteristics and high-yield cultivation techniques of Liaojing 9. *Bull. Agric. Sci. Tech.* 164-166 (2011).

16. Jiang, P., Xie, X., Huang, M., Zhou, X., Zou, Y. Yield formation characteristics and nitrogen use efficiency of super rice under different yield levels. *National Summit Forum on Multi-cropping and Grain and Oil Safety* (2015).

17. Wang, D. *Climatic characters and varietal condition of high-yielding early, middle and late season rice in the middle and lower reaches of the Yangtza River*, Huazhong Agricultural University, (2016).

18. Feng, Y. *et al.* Preliminary approach on adaptability of ORYZA2000 model for single cropping rice in Guiyang region. *Chin. Agric. Sci. Bull.* (2012).

19. Li, J. *Effects of ecological conditions and transplanting density on the rice population characteristics, yield and quality*, Sichuan Agriculture University, (2013).

20. Wang, D. *Effects of integrated crop management, climatic conditions, and soil fertility on dry matter production and yield formation of double-season rice and the related mechanism*, Huazhong Agricultural University, (2016).

21. Hu, Z. *Effect of Seedling Age on Premature Heading and Yield in Rice*, Huazhong Agricultural University, (2015).

22. Tian, G. *Management practices for achieving high yield and high resource use efficiency of rice in rice-rice-rape cropping system*, Huazhong Agricultural University, (2014).

23. Lin, H. *et al.* Effects of row-spacing on yield of super-high-yielding early and late rice. *Chin. J. Rice Sci.* **25**, 79-85 (2011).

24. Liu, L. *et al.* Differences in yield response to nitrogen fertilizer among rice cultivars and their relationship with root morphology and physiology. *Acta Agron. Sin.* **40**, 1999-2007 (2014).

25. He, H., Zeng, Y., Jia, W., Pan, X., Shi, Q. Effects of planting density on radiation use and grain yield of Tianyouhuazhan. *Hybrid Rice* **30**, 65-70 (2015).

26. Qin, P. *et al.* Preliminary study on productive adaptability of super rice Teyou 582 in different ecological sites of Guangxi. *J. Southern Agric.* **45**, 575-579 (2014).

27. Huang, M. *et al*. Agronomic performance of late-season rice in South China. *Plant Prod. Sci.* **21**, 32-38, (2018).

28. van Wart, J., Kersebaum, K. C., Peng, S., Milner, M., Cassman, K. G. Estimating crop yield potential at regional to national scales. *Field Crops Res.* **143**, 34-43 (2013).

29. Zhang, T., Yang, X., Wang, H., Li, Y., Ye, Q. Climatic and technological ceilings for Chinese rice stagnation based on yield gaps and yield trend pattern analysis. *Glob. Chang. Biol.* **20**, 1289-1298 (2014).

30. Chen, Y., Wang, P., Zhang, Z., Tao, F., Wei, X. Rice yield development and the shrinking yield gaps in China, 1981–2008. *Reg. Environ. Change* **17**, 2397-2408 (2017).

31. Lv, Z. *et al.* Climate change impacts on regional rice production in China. *Clim. Change* **147**, 523-537 (2018).

32. Agustiani, N. *et al.* Simulating rice and maize yield potential in the humid tropical environment of Indonesia. *Eur. J. Agron.* **101**, 10-19 (2018).

33. Laborte, A. G. *et al.* Rice yields and yield gaps in Southeast Asia: Past trends and future outlook. *Eur. J. Agron.* **36**, 9-20 (2012).

34. Stuart, A. M. *et al.* Yield gaps in rice-based farming systems: Insights from local studies and prospects for future analysis. *Field Crops Res.* **194**, 43-56 (2016).

35. Espe, M. B. *et al.* Yield gap analysis of US rice production systems shows opportunities for improvement. *Field Crops Res.* **196**, 276-283 (2016).

36. van Oort, P. A. J. *et al.* Assessment of rice self-sufficiency in 2025 in eight African countries. *Glob. Food Sec.* **5**, 39-49 (2015).

37. Tao, F., Zhang, S., Zhang, Z., Rötter, R. P. Temporal and spatial changes of maize yield potentials and yield gaps in the past three decades in China. *Agric., Ecosyst. Environ.* **208**, 12-20 (2015).

38. Grassini, P. *et al.* How good is good enough? Data requirements for reliable crop yield simulations and yield-gap analysis. *Field Crops Res.* **177**, 49-63 (2015).

39. Chen, X. *et al.* Producing more grain with lower environmental costs. *Nature* **514**, 486 (2014).

40. Huang, J., Wei, W., Cui, Q., Xie, W. The prospects for China's food security and imports: Will China starve the world via imports? *J. Integr. Agric.* **16**, 2933-2944 (2017).

41. Xiong, W., Conway, D., Lin, E., Holman, I. Potential impacts of climate change and climate variability on China’s rice yield and production. *Clim. Res.* **40**, 23-35 (2009).

42. Ye, L. et al. Climate change impact on China food security in 2050. Agron. Sustain. Dev. **33**, 363-374 (2013).

43. Xu, S., Li, G., Li, Z. China agricultural outlook for 2015-2024 based on China Agricultural Monitoring and Early-warning System (CAMES). *J. Integr. Agric.* **14**, 1889-1902 (2015).

44. OECD-FAO (the Organization for Economic Cooperation and Development and Food and Agricultural Organization of the United Nations). OECD-FAO Agric. Outlook 2018–2027. https://stats.oecd.org

45. USDA (the United States Department of Agriculture). International Long-Term Projections to 2025. https://www.ers.usda.gov/data-products/international-baseline-data/ (2016).
